# Eléphantiasis du membre inférieur gauche

**DOI:** 10.11604/pamj.2018.29.95.14838

**Published:** 2018-01-31

**Authors:** Youssef Zemmez, Mohammed Boui

**Affiliations:** 1 Service de Dermatologie, Hôpital Militaire d'Instruction Mohamed V, Rabat, Maroc

**Keywords:** Eléphantiasis, chirurgie, traitement préventif, Elephantiasis, surgery, preventive treatment

## Abstract

We report the case of a 48-year old female patient with a pathological history of chronic venous insufficiency which required a surgical treatment 5 years before, presenting in the Department of Dermatology with increased left lower limb evolved over the past 4 years. Clinical examination showed increased lower limb, compared to the contralateral limb, with surgical scar at the level of the external face of the left leg and some papillomatous lesions involving the entire left foot and covering the five left toes. The diagnosis of post-traumatic lymphedema (post-surgical) was retained. Treatment was based on compression stocking, lymphatic drainage as well as on a preventive treatment including benzathine benzylpenicillin: 1.2 MU every three weeks. Lymphedema is caused by a malfunction of the lymphatic system responsible for lymph stasis in interstitial tissues. This results in an increased limb volume. All the anatomical regions which contain lymphatic fluids can be affected by lymphedema, but it mainly occurs in the limbs. Lymphedema of the lower limbs can be classified as either primary or secondary. End-stage lymphedema is also known as elephantiasis.

## Image en médecine

Patiente âgée de 48 ans, ayant comme antécédents pathologiques une insuffisance veineuse chronique ayant nécessité un acte chirurgical depuis 05 ans, qui a consulté en dermatologie pour augmentation de la taille du membre inférieur gauche évoluant progressivement depuis 04 ans. L'examen clinique a objectivé un membre inférieur augmenté de taille par rapport au membre controlatéral, avec une cicatrice chirurgicale au niveau de la face externe de la jambe gauche, des lésions papillomateuses intéressant la totalité du pied gauche et couvrant les cinq orteils gauches. Un lymphœdème post-traumatique (post-chirurgical) a été retenu. Un traitement à base de bas de contention, drainage lymphatique a été prescrit ainsi qu'un traitement préventif a été préconisé incluant le benzathine benzyle pénicilline : 1,2 MU tous les trois semaines. Le lymphœdème est la conséquence d'un dysfonctionnement du système lymphatique responsable d'une stase de la lymphe dans les tissus interstitiels, se traduisant par l'augmentation de volume du membre. Toutes les régions anatomiques pourvues de lymphatiques peuvent être atteintes de lymphœdème, mais l'atteinte des membres est la plus fréquente. Au niveau des membres inférieurs, on distingue les lymphœdèmes primaires et secondaires. L'éléphantiasis correspond au stade ultime de l'évolution du lymphœdème.

**Figure 1:**
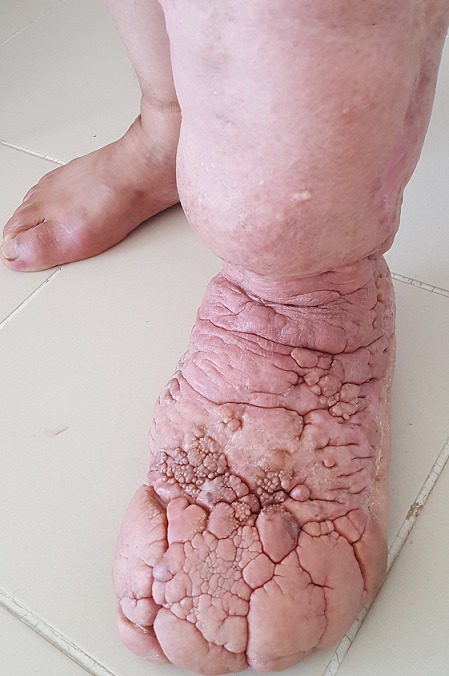
Eléphantiasis du membre inférieur gauche

